# Inhibitory NK Receptor Recognition of HLA-G: Regulation by Contact Residues and by Cell Specific Expression at the Fetal-Maternal Interface

**DOI:** 10.1371/journal.pone.0008941

**Published:** 2010-01-28

**Authors:** Tsufit Gonen-Gross, Debra Goldman-Wohl, Berthold Huppertz, Dikla Lankry, Caryn Greenfield, Shira Natanson-Yaron, Yaron Hamani, Ronit Gilad, Simcha Yagel, Ofer Mandelboim

**Affiliations:** 1 The Lautenberg Center for General and Tumor Immunology, Hebrew University-Hadassah Medical School, Jerusalem, Israel; 2 Department of Obstetrics and Gynecology, Hadassah University Hospital, Mount Scopus, Jerusalem, Israel; 3 Institute of Cell Biology, Histology, and Embryology, Medical University of Graz, Graz, Austria; Centre de Recherche Public de la Santé (CRP-Santé), Luxembourg

## Abstract

The non-classical HLA-G protein is distinguished from the classical MHC class I molecules by its expression pattern, low polymorphism and its ability to form complexes on the cell surface. The special role of HLA-G in the maternal-fetal interface has been attributed to its ability to interact with specific receptors found on maternal immune cells. However this interaction is restricted to a limited number of receptors. In this study we elucidate the reason for this phenomenon by comparing the specific contact residues responsible for MHC-KIR interactions. This alignment revealed a marked difference between the HLA-G molecule and other MHC class I molecules. By mutating these residues to the equivalent classical MHC residues, the HLA-G molecule regained an ability of interacting with KIR inhibitory receptors found on NK cells derived either from peripheral blood or from the decidua. Functional NK killing assays further substantiated the binding results. Furthermore, double immunofluorescent staining of placental sections revealed that while the conformed form of HLA-G was expressed in all extravillous trophoblasts, the free heavy chain form of HLA-G was expressed in more distal cells of the column, the invasion front. Overall we suggest that HLA-G protein evolved to interact with only some of the NK inhibitory receptors thus allowing a control of inhibition, while permitting appropriate NK cell cytokine and growth factor production necessary for a viable maternal fetal interface.

## Introduction

The immune environment at the maternal fetal interface has seemingly paradoxical roles. On the one hand the maternal immune system must be active and vigilant to prevent bacterial or viral infection of the placenta and developing fetus. On the other hand, the maternal immune cells must not attack the semiiallogenic fetal cells. This interaction is further complicated by the fact that extravillous trophoblasts, cells of fetal origin, invade and migrate into the maternal tissues and spiral arteries and are found in close contact with maternal immune cells. One of the crucial factors to be considered in this special environment is the MHC status of trophoblast cells as these molecules can act as ligands for uterine immune cells, including T cells, NK cells and myelomonocytic cells [1]. The trophoblast cells do not express classical MHC class I and II molecules, except for a low levels of HLA-C [2], [3]. In contrast, the invasive trophoblasts express non-classical MHC class I molecules of which the most extensively studied is HLA-G. This molecule displays many unique features such as low polymorphism, a truncated cytoplasmic tail and restricted distribution to the extravillous cytotrophoblasts [4], [5], [6]. The restricted expression of HLA-G in the placenta where classical MHC class I molecules are repressed, is thought to play a pivotal role in the immunoprotection of the semiallogenic embryo [7], [8]. Indeed, following implantation, the pregnant uterus is remodeled as a site of innate immunity where specialized NK cells termed decidual NK (dNK) comprise more than 40% of the entire cell population in the decidua [9], [10], [11]. These dNK exhibit different phenotypic characteristics and functional abilities compared with the NK population found in the peripheral blood [12], [13] and their number in the decidua is progressively diminished from mid-gestation onwards [14].

NK cells possess a combination of activating and inhibitory receptors [15]. Three major inhibitory NK receptors are found on peripheral as well as on decidual NK cells: the CD94/NKG2 heterodimers which recognize the HLA-E molecule loaded with MHC class I signal peptide [16], [17], the Leukocyte Ig like receptor (LIR) family which recognizes various MHC class I molecules [18] and the killer Ig-like receptor (KIR) family which recognize mostly HLA-C proteins[19]. The KIR binding specificity is largely determined by the amino acid at position 80 of HLA-C [20]. Group 1 HLA-C (HLA-C1) allotypes, have an asparagine residue at position 80 conferring recognition by KIR2DL2 and KIR2DL3. Whereas group 2 HLA-C (HLA-C2) allotypes, with lysine at position 80, are recognized by KIR2DL1 [21], [22]. Variegated expression of these receptors leads to a repertoire of HLA specificities within any individual's NK cell population [23] and expression of a particular KIR on all NK cells might lead to immune deficiency [24]. Although dNK cells express a variety of these receptors, only two receptors are relevant in the context of HLA-G recognition by NK cells; KIR2DL4 and LIR-1 [25], [26], [27], [28], [29]. The necessity however of KIR2DL4 for reproductive success has been questioned [27]. Upon MHC class I engagement LIR-1 mediates a negative signal by its immune receptor tyrosine-based inhibitory motifs in the intracellular domain [30], [31]. This receptor shows an overall high affinity to HLA-G over other MHC class I molecules due to an avidity effect of the LIR-1 receptor to the HLA-G molecules, formed as a result of HLA-G disulfide-bound complexes [28], [29], [32], [33], [34], [35]. This efficient binding facilitates the inhibitory signaling of NK cells through the LIR-1 receptor.

As mentioned above, during placentation, the decidua is infiltrated with the distinctive decidual NK cell population which expresses a variety of receptors known to recognize MHC class I molecules [15]. A key question that emerges is the restricted pattern of HLA-G interaction with dNK cell receptors. While this molecule targets only two known receptors on dNK cells, it is not involved in inhibition through other NK inhibitory receptors. This specificity is especially interesting considering the novel function recently demonstrated for dNK as cytokine secretors rather than strictly cytolytic executors [36].

HLA-G is found on the cell surface of extravillous trophoblasts in both the free heavy chain (FHC) form and the B2 microglobulin bound conformed form. The FHC form of HLA-G is not recognized by LIR-1 yet in in-vitro experiments it interferes with LIR-1 binding to the conformed form thus attenuating immune inhibition [29]. We now investigated, in placental sections, whether the localization of the FHC and conformed forms of HLA-G may be indicative of a natural mechanism modulating LIR-1 inhibition. This modulation may prevent over inhibition, thus allowing for appropriate growth factor expression necessary for development of the environment of the placental bed.

In the present study we compared the specific KIR contact residues found in classical MHC class I to that of HLA-G. We noticed that the HLA-G protein is different from classical MHC class I molecules in three contact residues. By converting HLA-G contact residues to that of HLA-C we discovered that the HLA-G molecule regains the ability to interact with NK inhibitory receptors of the KIR family. This interaction is functional and leads to inhibition of peripheral and decidual NK killing. Overall we suggest that HLA-G evolved to interact only with some of the inhibitory NK receptors to prevent an overwhelming inhibitory environment in the decidua which could lead to inadequate constructive signals essential to a proper development of the embryo.

## Materials and Methods

### Cells, Abs and Fusion Proteins

The cell lines used in this work are the MHC class I- negative EBV-transformed B cell line 721.221 (221) and 221 transfectants [20]. Primary NK cells were isolated from PBLs using the human NK cell isolation kit and the autoMACS instrument (Miltenyi Biotec). NK cells were kept in culture as described previously [20]. All mAbs used in this work were generated in mice, including W6/32 (IgG2a), directed against class I MHC molecules, anti-HLA-G mAb MEM-G/09 (IgG1), anti-CD85J/LIR-1 mAb- HPF1 (IgG1), anti KIR2DL1 mAb HP3E4 (IgM) (a kind gift from M. Lopez-Botet, DCEXS Universitat Pompeu Fabra, Spain), and anti- KIR2DL2 mAb GL183(IgG1).

The Ig fusion proteins used in this work are LIR 1-Ig, KIR2DL1-Ig, KIR2DL2-Ig, KIR2DS2-Ig, KIR2DS2 KYK/KFK-Ig (mutation generated by PCR as described below using the following primers: 5′KYK/KFK ctt ctg cac aga gag ggg ttt aag gac act ttg cac ctc att 3′KYK/KFK aat gag gtg caa agt gtc ctt aaa ctt ccc ctc tct gtg cag aag). Briefly, the sequence encoding the extracellular portion of the receptor was amplified by PCR from cDNA isolated from human NK clones. These PCR-generated fragments were cloned into a mammalian expression vector, containing the Fc portion of human IgG1. The construct was transfected into COS-7 cells, and the protein produced was purified using protein G column as described in [37], [38].

### Flow Cytometry

Cells were stained either with mAb or Ig fusion proteins. Second reagents were FITC-conjugated F(ab′)_2_ goat anti-mouse IgG (ICN Biomedicals) or the PE-conjugated F(ab′)_2_ goat anti-human Fc (Jackson ImmunoResearch Laboratories) directed against Ig fusion proteins. Monoclonal Abs were used at a final concentration of 2 µg/ml, and Ig fusion proteins at 50 µg/ml. The staining procedure was as follows: 50,000 cells were washed once in FACS medium (1x PBS, 0.5% BSA, and 0.05% NaN3) and then incubated in 100 µl of FACS medium containing either mAb or Ig fusion proteins for 1 or 2 h on ice (4°C), respectively. Incubations were performed in 96 U-shaped plates (Nunc). Cells were then washed twice in FACS medium and incubated on ice for 1 h with the appropriate second reagents. Following the incubation, cells were washed twice, resuspended in 200 µl of FACS medium, and analyzed on a FACSCalibur flow cytometer (BD Biosciences).

### Generation of 721.221 Cells Expressing Mutated HLA-G Molecule

For the generation of the mutated HLA-G protein we used a site-directed mutagenesis technique which utilizes the ability of *Dpn I* endonuclease specific to methylated and hemimethylated DNA to digest the parental DNA template and to select for mutation-containing synthesized DNA. The point mutations were performed on a PCDNA3 vector cloned with the HLA-G protein using the following primers: 5′M76V cac gca cag act gac aga gtg aac ctg cag acc ctg cgc ggc tac; 3′M76V gta gcc gcg cag ggt ctg cag gtt cac tct gtc agt ctg tgc gtg; 5′Q79R cac gca cag act gac aga atg aac ctg cgg acc ctg cgc ggc tac; 3′Q79R gta gcc gcg cag ggt ccg cag gtt cat tct gtc agt ctg tgc gtg; 5′N151R aag cgc aag tgt gag gcg gcc aga gtg gct gaa caa agg aga gcc; 3′N151R ttc gcg ttc aca ctc cgc cgg tct cac cga ctt gtt tcc tct cgg; 5′T80N act gac aga atg aac ctg cag aac ctg cgc ggc tac tac aac cag; 3′T80N ctg gtt gta gta gcc gcg cag gtt ctg cag gtt cat tct gtc agt; 5′T80K act gac aga atg aac ctg cag aag ctg cgc ggc tac tac aac cag; 3′T80K ctg gtt gta gta gcc gcg cag ctt ctg cag gtt cat tct gtc agt; 5′M76A+ Q79R cac gca cag act gac aga gtg aac ctg cgg acc ctg cgc ggc tac; 3′M76A+ Q79R: gta gcc gcg cag ggt ccg cag gtt cac tct gtc agt ctg tgc gtg; 5′T80N+ M76A+ Q79R act gac aga gtg aac ctg cgg aac ctg cgc ggc tac tac aac cag; 3′T80N+ M76A+ Q79R ctg gtt gta gta gcc gcg cag gtt ccg cag gtt cac tct gtc agt; 5′T80K+ M76A+ Q79R act gac aga gtg aac ctg cgg aag ctg cgc ggc tac tac aac cag; 3′T80K+ M76A+ Q79R ctg gtt gta gta gcc gcg cag ctt ccg cag gtt cac tct gtc agt. Following amplification, the product is treated with *DpnI* which digest the original plasmid (originated in E.coli dam^+^ strain and therefore methylated) and then transformed into bacterial competent cells.

### Isolation of Human NK Subsets

The institutional board of Hadassah organization approved the use of decidual and placental waste material from elective pregnancy termination procedures, according to the principles of the Helsinki Declaration. In this study, lymphocytes from parts of decidua basalis and parietalis were used as previously described [39]. Peripheral blood lymphocytes were isolated from different healthy donors using Ficoll gradients. Isolation of NK cells was performed by using NK isolation kit II (Miltenyi Biotec), according to manufacturer's instructions.

### Cytotoxicity Assays

The cytotoxic activity of NK cells against various targets was assayed in 5-h [S35] Met release assays, as described previously [20].

### HLA-G Double Staining

The study was approved by the ethical committee of the Medical University of Graz. Informed written consent in which it was stated that we are allowed to use termination material to study cellular and molecular interactions in the feto-matenal contact zone was obtained from the patients. Three first trimester placentas between weeks 8 and 11 of gestation were obtained from pregnancy terminations for psychosocial reasons. Tissue samples were fixed and paraffin embedded using the HOPE technique, as described by Blaschitz et al. (2008). In brief, small pieces of tissues were fixed in ice cold (2°C) HOPE I solution for 1 d to 3 d. Samples were transferred into ice-cold HOPE II solution (diluted 1∶1000 in acetone) for 2 h, followed by three 2 h steps of ice cold acetone dehydration. Tissues were soaked in low temperature paraffin (melting point of 52–54°C) overnight and embedded.

5 µm sections were mounted on Superfrost Plus slides (Menzel-Glaeser) and deparaffinized according to the manufacturer's instructions (DCS, Hamburg, Germany). Sections were blocked for 7 min with Ultra V Block (Lab Vision/Thermo Fisher scientific, USA) containing 10% human AB-serum. Slides were incubated with the primary antibody, anti-HLA-G (clone 4H84, Exbio, Prag, 1∶3000) in antibody diluent (Dako, USA) for 30 min at RT. After washing with PBS a goat anti mouse IgG conjugated with Alexa Fluor 555 (Invitrogen, Molecular Probes, Eugene, Oregon, USA) was used (1∶200; 30 min, RT). Following a further washing step with PBS, a second anti-HLA-G antibody was applied (clone MEM-G9 conjugated with Alexa Fluor 488, 10 µg/ml) for 60 min. Slides were washed with PBS and nuclei stained with DAPI (1∶2000; Invitrogen) for 5 min. Slides were mounted with ProLong Gold antifade reagent (Invitrogen). Fluorescence microscopy was performed using a Leica DM 6000B microscope and an Olympus DP 72 Camera.

## Results

### HLA-G Contains Different KIR Contact Residues Compared with Other MHC Class I Molecules

**Figure 1:**
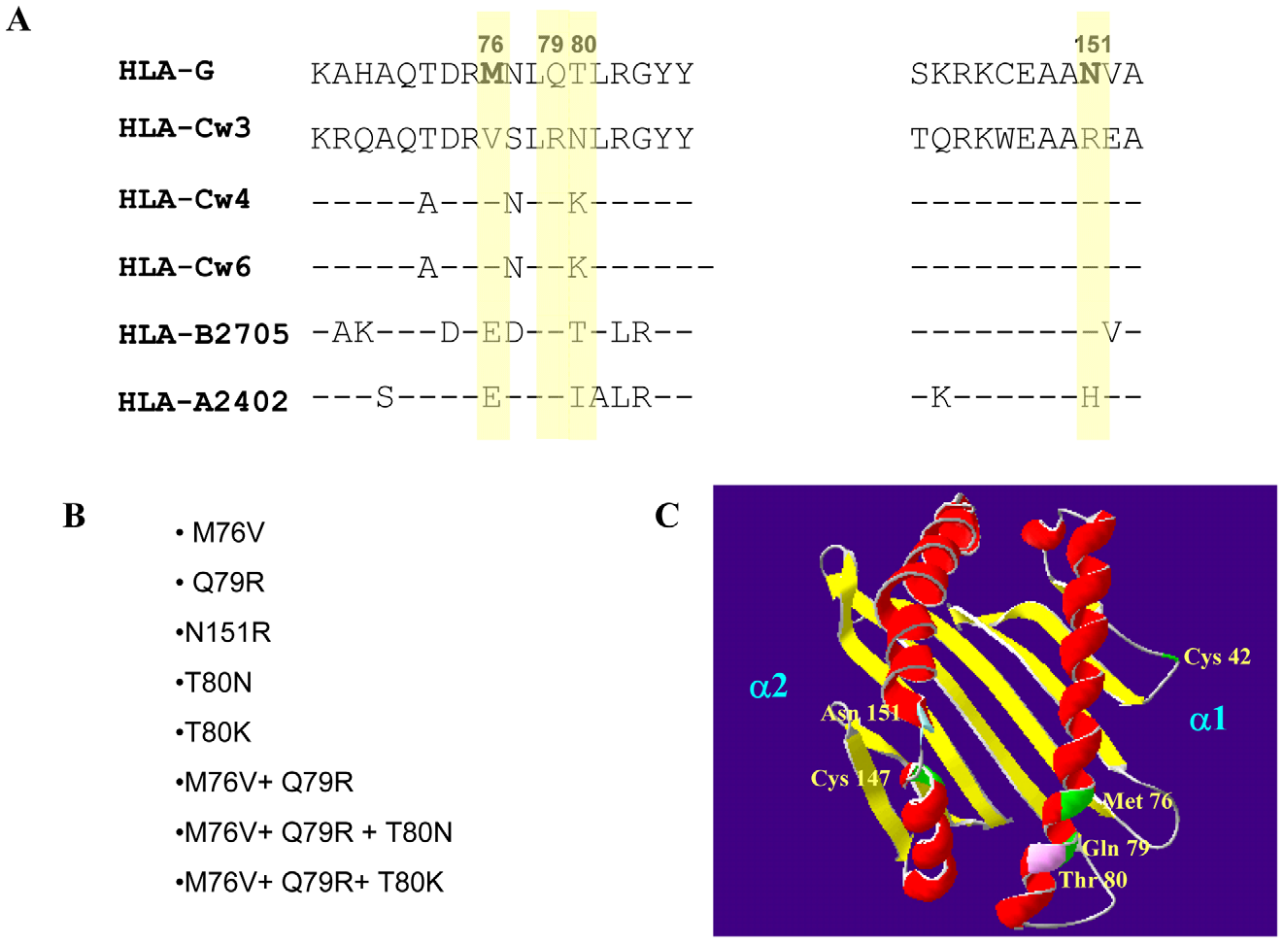
HLA-G is markedly different from HLA-C and other selected MHC class I molecules in the contact residues between KIR and HLA-C. (A) Sequence alignment between representative MHC class I molecules of the HLA-A, B and C sub-classes and HLA-G in the binding interface with KIR inhibitory receptors. The HLA-G residues that were selected for site-directed mutagenesis are shown in bold and highlighted in yellow. The sequences are shown for two regions (positions 68–85 and 143–153). Conserved residues are indicated by dashes. (B) A ribbon diagram of the crystal structure of HLA-G with the contact residues superimposed. Cys 42 and Cys 147 which form disulfide bridges for the formation of HLA-G complexes and the contact residues that were mutated are indicated. Domains α1 and α2 are also indicated. This backbone modeling of the HLA-G molecule was generated using Swiss-PDB viewer v3.7 software. (C) A list of the single, double and triple mutations which were performed in the HLA-G molecule.

Although dNK cells express various inhibitory receptors of the killer-immunoglobulin-like receptors (KIR) and the C-type lectin heterodimer family (CD94/NKGs), only two NK receptors predominantly recognize the HLA-G molecule; LIR-1 (of the LIR or ILT family) [40], [41] and KIR2DL4 [42], [43]. Among these two receptors, the KIR2DL4 binds HLA-G with a very low affinity and the interaction with HLA-G is probably more significant with regard to its soluble product which leads to the secretion of a angiogenic factors [25]. To understand why this specificity has evolved we initially examined the specific contact residues between the HLA-C molecule and the KIR receptors and compared them to the same residues in the HLA-G molecule (Fig. 1A). Inhibitory KIR2D receptors are divided into two families based on their specificities for different HLA-C allotypes and residue 80 of HLA-C has been implicated to mediate this specificity [20]. While, KIR2DL1 exhibits C2 specificity and recognizes HLA-C alleles with Lys 80 (e.g. HLA-Cw4 and HLA-Cw6), KIR2DL2 has C1 specificity and recognizes alleles with Asn 80 (e.g. Cw3). Although the KIR/HLA-C interface possess more residues, we focused on four contact residues; Met76, Gln79, Asn151 and Thr 80 (Fig. 1b). These amino acid residues were previously shown to participate either in KIR2DL1 or KIR2DL2 binding to the HLA-Cw4 and HLA-Cw3 molecules respectively [21], [22]. Sequence alignment of the HLA-G and selected MHC class I molecules in the binding region of the KIR inhibitory receptors revealed that HLA-G differs from the HLA-C molecules in these specific contact residues (highlighted in yellow).To understand the role of the contact HLA-G residues in HLA-G recognition by NK cells we performed an extensive site-directed mutagenesis which is listed in figure 1B. In every mutant we replaced the amino acid residue of HLA-G with the equivalent residue in the HLA-C molecule. In addition we constructed a double mutant of HLA-G at residues 76 and 79 and a triple mutant which contains the double mutation and another mutation in threonine 80 either to aspargine or to lysine (which mimics the KIR2D binding site to HLA-C1 or C2 allotypes respectively). A graphic view of the selected contact residues is also shown in a carbon diagram of the HLA-G molecule (Fig. 1C).

### HLA-G Is Recognized by the Inhibitory KIR-Ig Fusion Proteins Only When It Is Mutated in Three Contact Residues

**Figure 2:**
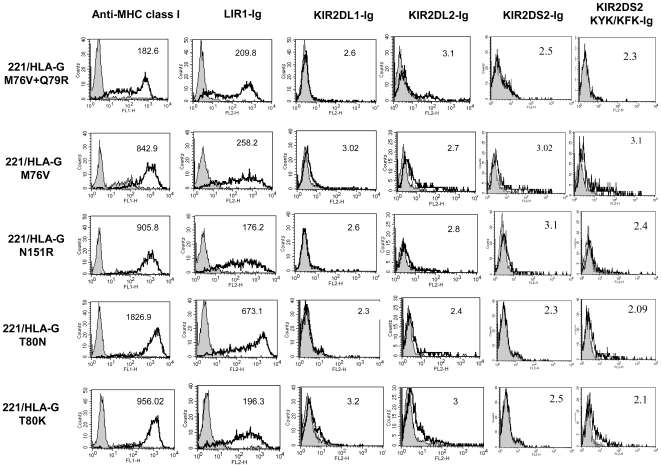
Binding of various fusion proteins to the mutated 221/HLA-G molecule is not affected by single or double mutations in the contact residues. 221\HLA-G mutated in the KIR-HLA-C contact residues were stained with various fusion proteins followed by secondary antibody staining. Gray histograms represent background secondary antibody staining. The numbers shown in each histogram indicate the median fluorescence intensity, MFI. Expression levels, were monitored with anti-MHC class I mAb (left panel). Shown is a representative experiment of at least three independent experiments.

**Figure 3:**
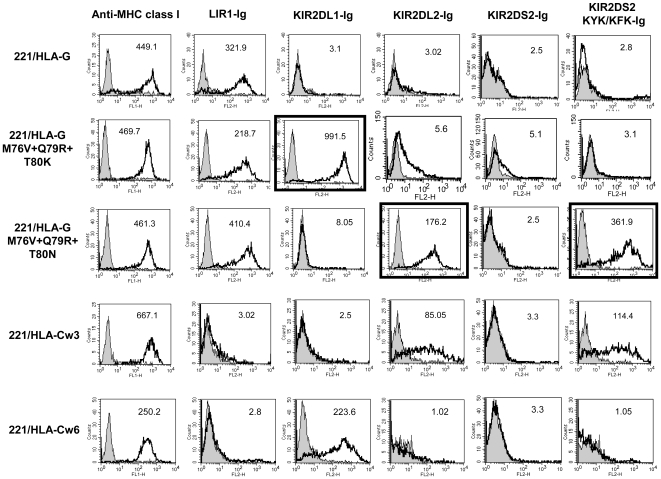
221/HLA-G mutated in three contact residues are recognized by KIR-Ig fusion proteins. Wild-type, triple mutated 221/HLA-G, 221/HLA-Cw3 and 221/HLA-Cw6 were stained with various fusion proteins followed by secondary antibody staining. Gray histograms represent background secondary antibody staining. For confirmation of expression level, cells were stained with anti-MHC class I mAb (left panel). Black frames emphasize the unique KIR-Ig binding to the 221/HLA-G triple mutants. Shown is a representative experiment of at least three independent experiments.

To study how the binding of NK inhibitory receptors is influenced by the mutations in the contact residues we stained the 221/HLA-G mutants with Ig-fusion proteins in which the extracellular portion of the receptor is fused to immunoglobulin G1 (IgG1), as described [38], [37]. As expected, binding of the LIR1-Ig which interacts with the HLA-G protein through the α3 domain is not dramatically affected by the mutations which are located in α1 and α2 portions of the molecule. However, when we tested the binding of receptors from the KIR family to the various mutants we noticed an interesting phenomenon. While the single or double HLA-G mutations (i.e. M76V+Q79R, M76V, Q79R, N151R, T80N, T80K, Fig. 2) were not stained by KIR2DL1-Ig or KIR2DL2-Ig, the triple HLA-G mutations demonstrated a different pattern of binding. The triple HLA-G mutant which mimics the binding site of the HLA-C1 allotypes (i.e. 221/HLA-G M76V+Q79R+T80N) was strongly stained with KIR2DL2-Ig (Fig. 3, bold black frame) but not with KIR2DL1-Ig. No staining was observed with the activating form of KIR2DL2 i.e. KIR2DS2–Ig, in agreement with previous publications demonstrating low affinity interactions between activating KIRs and MHC class I molecules [38], [44], [45]. However, when the contact residues in KIR2DS2 were mutated to contain these of KIR2DL2 (KYK/KFK) efficient binding to HLA-G was observed (Figure 3). 221/HLA-Cw3 which serves as a positive control interacts with both KIR2DL2-Ig and KIR2DS2 KYK/KFK-Ig (figure 3). Staining of the triple mutant which mimics the binding site of the HLA-C2 allotypes (i.e. 221/HLA-G M76V+Q79R+T80K) resulted in a strong binding of the KIR2DL1-Ig (Fig. 3, bold black frame). As expected, the positive control. 221/HLA-Cw6 interacts with KIR2DL1-Ig only (Figure 3). To exclude any possible influence of the mutations on the protein structure and expression level we stained the various 221/HLA-G mutants with two conformational-dependent anti-MHC class I mAbs and observed staining of all mutants (Figures 2 and 3 left panel and data not shown).

### The Three Critical Contact Residues Are Functionally Important in NK Mediated Inhibition through the Relevant Receptors

**Figure 4:**
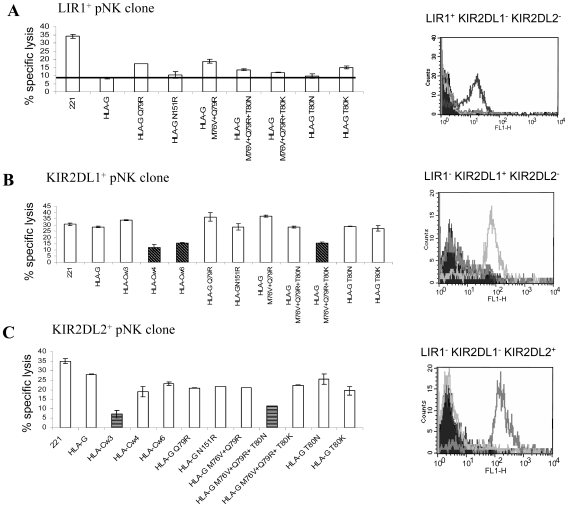
The triple HLA-G contact residues mutants affect peripheral NK clones killing activity. Various S^35^-labeled cells were incubated with (A) LIR-1^+^, (B) KIR2DL1^+^ or (C) KIR2DL2^+^ peripheral NK clone in effector to target ratio (E∶T) of 4∶1. The expression of a particular NK receptor on each of the clones is presented in A–C. Shown is one representative experiment out of four performed.

To further ascertain the binding results and to determine whether these results are functionally significant we conducted killing assays using peripheral NK clones which express one of the NK inhibitory receptors, LIR-1 (Fig. 4A), KIR2DL1 (Fig. 4B) and KIR2DL2 (Fig. 4C). As targets we used various 221 transfectants including wild-type HLA-G, all of the HLA-G mutants, HLA-Cw3, HLA-Cw4 and HLA-Cw6.

In correlation with the binding results, NK clones expressing the LIR-1 receptor are inhibited by all of the various HLA-G mutants compared to 221 (Fig. 4A). However, when the 221/HLA-G mutants are assayed with KIR2DL1^+^ NK clones, only the triple mutant which mimics the binding site of the HLA-C2 allotypes (i.e. 221/HLA-G M76V+Q79R+T80K) inhibits the killing, in a similar manner to that observed with 221/HLA-Cw4 and 221/HLA-Cw6 (Fig. 4B, dark bars). When the various HLA-G mutants were incubated with KIR2DL2^+^ NK clones only the triple mutant which mimics the binding site of the HLA-C1 allotypes (i.e. 221/HLA-G M76V+Q79R+T80N) inhibits the killing similar to that of 221/HLA-Cw3 (Fig. 4C, dark bars).

**Figure 5:**
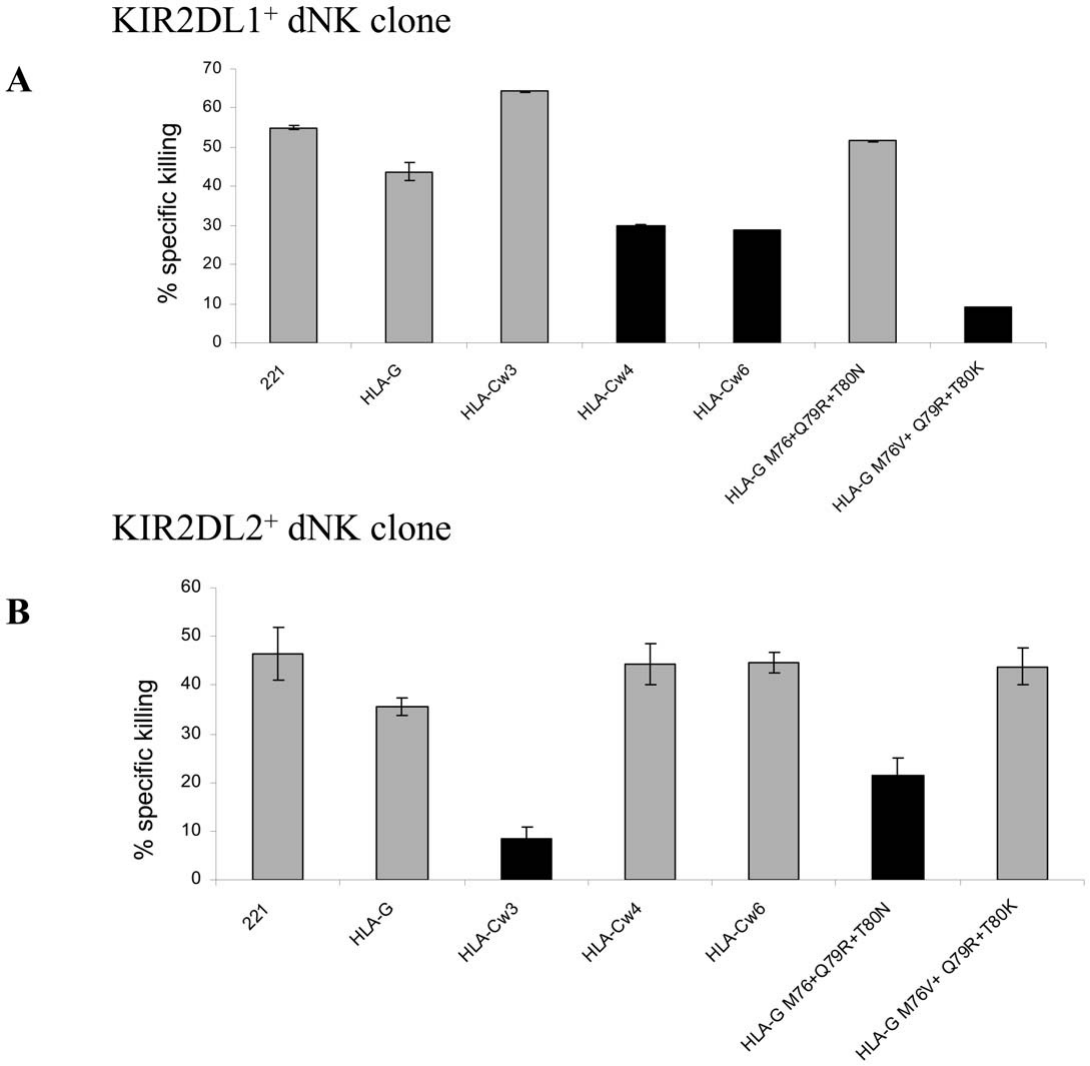
The triple HLA-G contact residues mutants affect decidual NK clones killing activity. Various S^35^-labeled 221 transfected cells were incubated with (A) KIR2DL1^+^ or (B) KIR2DL2^+^ decidual NK clone in effector to target ratio (E∶T) of 5∶1. Shown is one representative experiment out of two performed.

We next investigated whether the same phenomenon will be reproduced also with decidual NK clones. Two representative decidual NK clones expressing the KIR2DL1 receptor (fig. 5A) or the KIR2DL2 receptor (fig. 5B) are presented in figure 5. As expected, a similar killing and inhibition pattern was observed in these decidual clones and only the HLA-G triple mutants inhibit the killing depending on the expression of the appropriate KIR receptor (Figure 5). Thus, we suggest that these three contact amino acid residues are critical to prevent a general inhibitory mechanism of NK in the decidua.

### The FHC and Conformed Forms of HLA-G Are Differentially Expressed on Extravillous Trophoblasts

**Figure 6:**
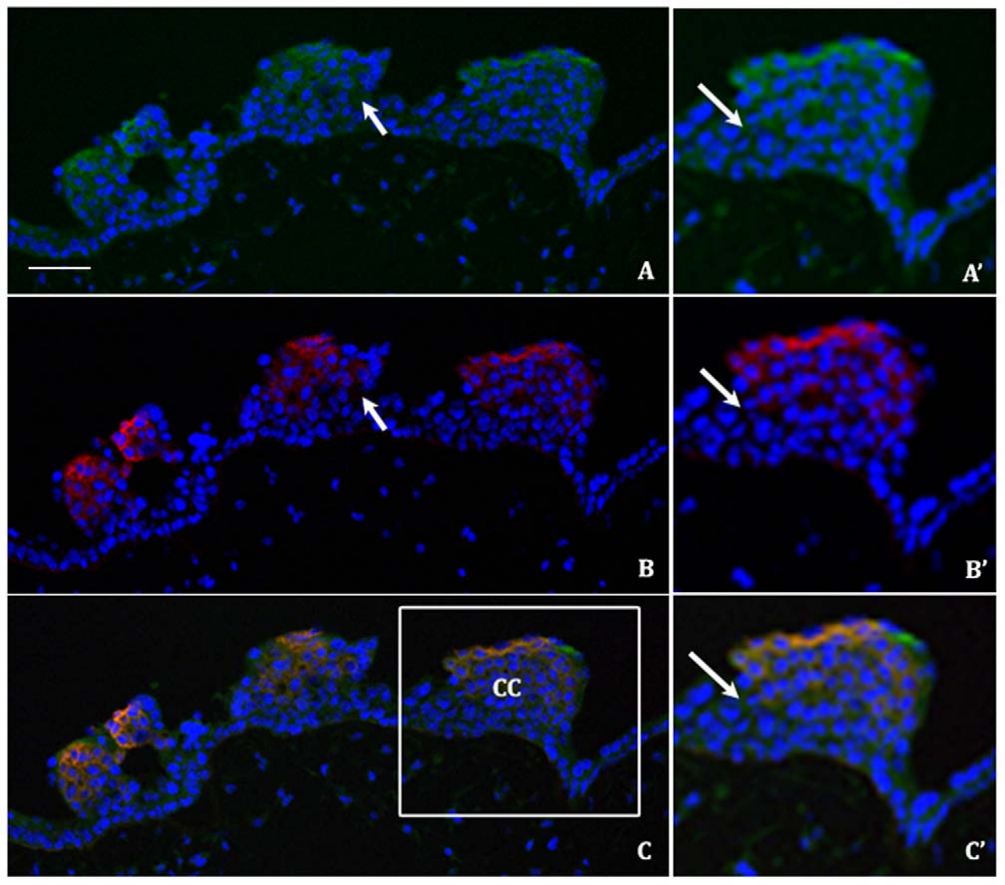
The conformed and FHC forms of HLA-G are differentially expressed in trophoblast cell columns. Double immunofluorescent staining of representative first trimester placental sections (week 10–11) with antibody MEM-G9 (A,A′) and 4H84 (B,B′) and the combined image (C,C′) (scale bar 50 µM). DAPI (blue), conformed HLA-G (green) and FHC HLA-G (red) staining is observed in the panels with the magnified inset (boxed area in image C) viewed in panels A′,B′,C′. Several areas where differential expression of conformed and FHC HLA-G are indicated by arrows. CC indicates a trophoblast cell column.

Extravillous trophoblasts form anchoring cell columns that serve to attach the placenta to the uterus. These invasive cells contact maternal NK cells when they migrate into the uterine tissue, the decidua, and progress through the first third of the myometrium, and the spiral arteries. We performed double immunofluorescent staining to determine if there is differential expression of the HLA-G FHC and conformed forms on extravillous trophoblasts. Interstingly, while the antibody (4H84) recognizes the FHC functions in both cryopreserved and formalin fixed paraffin embedded sections, the antibody that recognizes the conformed HLA-G (MEM-G9) functions only on the cryopreserved sections. The conformed HLA-G is found in the cell column and includes cells more proximal to the floating villous (figure 6A,A′), whereas the FHC form of HLA-G (figure 6B,B′) is expressed more distally on the invasive front of the trophoblast cell column. The combined figure shows that conformed HLA-G form are present in areas which are negative for FHC staining (Figure 6C,C′).

## Discussion

The importance of NK cells in regulating processes at the unique maternal-fetal environment has long been recognized in the first half of pregnancy, based on the massive enrichment of maternal decidua with NK cells. These dNK cells are characterized by distinctive markers and abilities which distinguish them from peripheral NK cells [13]. They are actively recruited to this area upon embryo implantation [9] and are found particularly in areas of infiltrating fetal trophoblast cells which invade the decidua [46]. These trophoblast cells express the non-classiacal MHC class I molecule HLA-G which plays a central role in immunosuppressing a large variety of immune cells [1]. An extensive research has been conducted on the ability of HLA-G to mediate NK cell inhibition [47]. However, emerging evidence support the idea that the function of HLA-G may not be solely in inhibition but rather in modulation of cytokine secretion from dNK cells [25], [48].

In this study we found differential site specific expression of the FHC form of HLA-G as compared to the conformed form of the molecule suggesting that trophoblast invasion and expression of the FHC form are linked. Post-transcriptional regulation of cell surface expression of HLA-G has been described as the HLA-G RNA is expressed throughout the trophoblast cell column but the HLA-G protein was found to be expressed only at the distal end of the column.

The antibody used in those experiments is now known to bind only the FHC form of the molecule. In agreement with these results we find that the FHC form of the molecule is distally expressed in the cell column but here we find that the conformed form is expressed more widely and proximal to the floating villous. This is also in agreement with the finding that TAP1, TAP2, tapasin and beta (2)-microglobulin are expressed similar to the conformed form of HLA-G throughout the cell column. As previously described, HLA-G FHC does not bind LIR-1 and may interfere with the conformed LIR-1 and HLA-G interaction. Furthermore, specific cytokine and growth factor production is inhibited when LIR-1 positive dNK cell clones are incubated with HLA-G transfectants. Thus it is possible that expression of the FHC HLA-G in the invasive trophoblasts may serve to attenuate LIR-1 inhibition and allow for the appropriate cytokine production by dNK necessary for pregnancy to succeed.

In this study we further tested the HLA-G properties by focusing on the interactions between HLA-G and NK inhibitory receptors. dNK cells express a diverse set of inhibitory receptors known to recognize MHC class I molecules [15]. HLA-G, which is the dominate MHC class I protein expressed on the trophoblasts, is however, able to contact with only two known receptors; one inhibitory LIR-1 and the second activating KIR2DL4.The KIR family have been present in species since at least 135 million years ago [51]. The *HLA-C* gene originated from a duplication of an ancestral *HLA-B*-like gene, which took place in an ancestor of humans and great ape species approximately 12 million years ago. The *HLA-A* and -*B* genes are much older, and orthologues have been described in Old World monkey species such as the rhesus macaque. The evolutionary history of the *HLA-G* gene is peculiar because, although great apes have an orthologue of HLA-G, presumably with a similar function [52], the Old World monkey equivalent has been inactivated [53]. Based on our observations it is tempting to suggest that HLA-G evolved out of the KIR-binding HLA pool and actively mutated its key KIR-binding residues.

To understand this limited recognition we conducted a sequence alignment between HLA-G and other MHC class I molecules and compared the four contact residues responsible for KIR-mediated recognition of MHC class I molecules. Indeed, the HLA-G is completely different from other MHC class I molecules in this binding interface (fig. 1A and B). By mutating these residues we restored the binding between the HLA-G protein and the KIR inhibitory receptors. The binding was dependent on three critical amino acid residues M76V, Q79R and T80K/N and was functional. In the HLA-C molecules the binding to a particular NK inhibitory receptor is determined by residue 80 [20].The two other contact residues at positions 76 and 79 are identical between the C1 and C2 groups [49]. However in HLA-G, the three contact residues at positions 76, 79 and 80 are different and thus interaction with all KIR receptors is prevented. Importantly, converting the contact residues of HLA-G to those found on HLA-C did not alter the binding to the LIR-1 inhibitory receptor. Thus, if inhibition of NK killing was the primary function of HLA-G, we would expect the contact residues in HLA-G to be similar to that of HLA-C. The fact that this is not the case and the fact that not only residue 80 but also residues 76 and 79 are different in HLA-G suggest for a deliberate evolutionary mechanism. What is the reason for such an unusual phenotype? What advantage offers the fact that HLA-G has such limited ability to bind KIRs?

Recently a gene linkage analysis showed that receptor-ligand combinations favoring dNK inhibition increased the likelihood of preeclampsia [50]. The molecular basis for this gene linkage may be the result of a shortage in NK-derived growth factors and chemokines for invading trophoblasts and decidual blood vessels as was lately shown by our group [36]. Thus, too much inhibition of NK cells is dangerous at the fragile fetal-maternal interface. Our results point to the same conclusion but from another point of view.

The HLA-G molecule due to its many unique features and its immuno-modulatory abilities is now well recognized to play a central role in mediating tolerance to the semi-allogenic fetus by the maternal immune system. However, recent articles shed new light on the interactions between the maternal immune system and the fetus. It is now established that these interactions have also a physiological function in regulating the development of the placenta, rather than represent solely a maternal immunological defense reaction against the allogenic fetus. Thus, the unique mechanism for HLA-G that enables a maternal NK discrimination and differentiated inhibition can offer HLA-G some advantages. For instance, the advantage of a monomorphic HLA-G∶HLA-G-receptor binding site compared to the HLA∶KIR binding families; the advantage of signaling pathways through LIR molecules sufficiently different from those of KIRs to specifically inhibit some dNK functions and not interfere with others;. However, when considering the unique role HLA-G possess we should remember that other receptors on dNK cells beside LIR-1 are able to interact with HLA-G as CD8, CD160 and KIR2DL4. By binding these receptors HLA-G may mediate inverse function of both pro- and anti-angiogenic properties resulting in a net effect to enable a proper uterine vascular remodeling.

Thus, as HLA-G^+^ trophoblast cells infiltrate the uterine mucosa, they deliver a pregnancy-specific signal to the local maternal NK cells and modify this unique environment in a way that enables a delicate protection to fetal tissues from the maternal immune system.

To conclude, Based on our mutational analysis in HLA-G and our in situ observation of FHC as compared to conformed HLA-G in placental tissue we suggest that too much inhibition by HLA-G is dangerous and that the specific interaction of HLA-G with only one inhibitory receptor LIR-1 is to generate a situation in which only a fraction of NK cells expressing LIR-1 are inhibited.

An interesting, still unanswered question in this regard is what so special about the LIR1-positive decidual NK cell subset.
